# Memory of Fictional Information: A Theoretical Framework

**DOI:** 10.1177/17456916231202500

**Published:** 2023-11-02

**Authors:** Pierre Gander, Kata Szita, Andreas Falck, Robert Lowe

**Affiliations:** 1 Department of Applied Information Technology, University of Gothenburg; 2 Trinity Long Room Hub Arts & Humanities Research Institute, Trinity College Dublin; 3 ADAPT Centre of Excellence for AI-Driven Digital Content Technology, Trinity College Dublin; 4 Department of Special Needs Education, University of Oslo

**Keywords:** memory, fiction, fictionality, fictional information, mechanisms, fiction-reality distinction

## Abstract

Much of the information people encounter in everyday life is not factual; it originates from fictional sources, such as movies, novels, and video games, and from direct experience such as pretense, role-playing, and everyday conversation. Despite the recent increase in research on fiction, there is no theoretical account of how memory of fictional information is related to other types of memory or of which mechanisms allow people to separate fact and fiction in memory. We present a theoretical framework that places memory of fiction in relation to other cognitive phenomena as a distinct construct and argue that it is an essential component for any general theory of human memory. We show how fictionality can be integrated in an existing memory model by extending Rubin’s dimensional conceptual memory model. By this means, our model can account for explicit and implicit memory of fictional information of events, places, characters, and objects. Further, we propose a set of mechanisms involving various degrees of complexity and levels of conscious processing that mostly keep fact and fiction separated but also allow information from fiction to influence real-world attitudes and beliefs: content-based reasoning, source monitoring, and an associative link from the memory to the concept of fiction.

People encounter more than factual information in everyday life. As a matter of fact, people spend large portions of their time consuming mediated content, such as movies, novels, and video games. Pretense and role-playing, experienced directly without a medium, are also common elements in people’s lives. Such activities can involve fiction or fictional information. Thus, fiction permeates many situations, and its purpose is not only entertainment but also education and socialization. Memory plays a key role in these activities for several reasons. First, it is only with the help of memory that people can recall and discuss their experiences of fiction. Second, experiences with fiction can influence attitudes, beliefs, and knowledge through explicit or implicit recollection—and hence the effects of fiction, both on individual and societal levels, significantly depend on memory.

Given the ubiquity of fictional information in everyday life, we propose that the notion of fictionality (whether a piece of information is understood as factual or fictional) is a necessary component for a general theory of human memory. With that in mind, in this article we show how memory of fiction is a distinct construct and how fictionality can fit into a conceptual model of general memory. Additionally, we examine a set of mechanisms of varying degrees of complexity and levels of conscious processing that allow for labeling information in memory as either factual or fictional. We aim to inform future studies that can tackle questions such as how memories of events from various mediated sources differ from one another (for instance, a novel or a virtual reality game), or how memory can influence involuntary behaviors (for instance, the fear of darkness after watching a horror movie). Future research may also reflect on the mechanisms we outline when studying the processes and shortcomings of the memory system when separating memories of, for example, real and role-played actions or when a terrorist attack described in a spy novel is incorrectly remembered as real.

There has been an increase in recent years in research on the cognitive processing of fiction, such as comprehension of media content or written texts (e.g., Abraham et al., 2008; Altmann et al., 2014; Consoli, 2018; Hartung et al., 2017; Jacobs & Willems, 2018; Triantafyllopoulos et al., 2021). Yet a unified theoretical view of how fiction is processed differently from fact has not been offered. Earlier research regarding the influence of fiction on real-world beliefs and knowledge considered source-monitoring processes as an explanation for how fact and fiction can be distinguished in memory (Butler et al., 2012; Gerrig & Prentice, 1991; Green & Brock, 2000; Marsh et al., 2003; Potts et al., 1989; Potts & Peterson, 1985). However, we argue that source monitoring by itself cannot account for this ability and that additional mechanisms need to be considered. Recent memory research attempts to place memory of fictional events in relation to a general theory of human memory (Marsh & Yang, 2020; Rubin, 2022; Rubin & Umanath, 2015; Yang et al., 2022). But these approaches are limited in the conceptual analysis of fictionality, and they address only event memory (not semantic memory) and explicit memory (not implicit memory).

In view of the identified gaps of previous research, we aim to (a) present memory of fiction as a distinct construct in relation to other cognitive phenomena, (b) provide a broader theoretical framework of how memory of fiction fits into a general picture of human memory, and (c) account for the underlying mechanisms involved in distinguishing memories of fact and fiction. Pursuing the first aim, we will show how memory of fiction differs from, for instance, episodic memory, episodic future thinking, false memories, and memory of false information. This establishes the importance of fictionality for memory research and the rationale for studying it as a distinct phenomenon. The second aim leads to demonstrating how fictionality can be integrated in a general theory of memory. By this, we conceptualize memory of fiction as a type of memory and present its properties. The specific memory model employed can also suggest new subcategories of memory of fiction warranting attention in future research. Last, the third aim helps to extend the scope of previous research into how fact and fiction are distinguished in memory and what cognitive mechanisms underpin this ability. Our theoretical framework offers a wider account in that it goes beyond source monitoring to explain the processes involved in the distinction between fact and fiction in memory. The characterization of the mechanisms that separate fact from fiction in memory can generate predictions with the goal of stimulating future empirical research.

We delimit our theoretical framework in the following ways. First, we develop the framework to understand how memory of fact and fiction differs, rather than to assess what cues or features observers, or researchers, use to classify fictionality in external sources. Second, the framework does not address the direct experience of fiction during comprehension. Third, as we focus on the fundamental question of fictionality, we omit the question of distinguishing between different fictional worlds (e.g., Skolnick & Bloom, 2006). Fourth, the framework is agnostic concerning the ontological status of fiction: It uses an ontological epistemological notion of fiction (Rapaport & Shapiro, 1995) and focuses on a cognitive agent’s understanding of fiction—or behavior in accordance with understanding it—instead of fiction as an absolute ontological concept. A key underlying assumption is that an agent attempts to distinguish between fact and fiction, and that this process is independent of the ontological questions of whether fact and fiction are socially constructed phenomena or objectively exist. Fifth, a neurological perspective is beyond the scope of this article, although linking our conceptual and information-processing account to neuroscience is a highly desirable future endeavor.

In the sections that follow, we will clarify the meaning of fictional information and discuss the cognitive separation of fact and fiction. Then we will review the connections of memory of fiction to other related phenomena. After discussing why existing theories are insufficient to account for memory of fictional information, we show how fictionality can be integrated in a conceptual memory model. We also describe a set of mechanisms involved in separating fact from fiction in memory. Finally, we offer a set of open questions to inspire future research.

## Memory of Fictional Information

### What is memory of fictional information?

We define *memory of fictional information* as memories formed as a result of a creative act of imagining some states of affairs, events, places, characters, or objects, with either internal or external origin, which are believed by the person remembering to be decoupled from the real world. *Decoupling* means that a piece of information is not intended to be evaluated against the real world and that real-world truth conditions and claims of existence are irrelevant (Consoli, 2018; Gander, 2005; Worth, 2004). Examples of external sources of fictional information are novels, plays, films, comic books (Busselle & Bilandzic, 2008; Yang et al., 2022), computer games, children’s pretense (Hopkins & Weisberg, 2017), role-playing (Seppänen et al., 2021), oral storytelling, and elements of everyday conversation (Brône & Oben, 2021; Chafe, 1994). An example of fictional information in everyday conversation is that people sometimes engage in joint pretense when using irony and humor. Additionally, fictional information could be generated internally by imagination, such as in daydreaming or a controlled intent to make something up.^1^ Fictional information is commonly associated with a narrative, but this is not a requirement (Consoli, 2018): It is possible to imagine, for instance, a fictional animal without it occurring as part of a narrative. Thus, our definition of fiction is not limited to fiction as part of a storytelling genre.

Narrative works and situations can contain both factual and fictional elements. For instance, a fictional story can involve the (real) president of the United States, real-world causal properties, or fictional characters’ psychological reactions that are taken from, and believed to be true to, the real world. We view a fictional work as a hierarchical organization of information. For instance, a novel can contain both factual and fictional events; fictional events may involve factual and fictional characters with factual and fictional characteristics. In this way, after a hierarchical breakdown, each piece of information, and consequently its memory representation, could be assigned a fictional status. The proportion of factual or fictional elements usually determines whether the work or situation as a whole is regarded as factual or fictional. This overall judgment of fictionality may be modulated by cultural conventions, such as genre conventions: For instance, a documentary is generally taken as factual, whereas a feature film is taken as fictional.

The following four cases are examples of memories of fictional information, which serve as the basis for the discussion in the rest of the article and as explananda for a theory of memory of fictional information: (a) the awareness that events, characters, and objects remembered from a novel about Harry Potter are fictional, whereas events, characters, and objects from a newscast are real; (b) the awareness that, in remembering a fantasy live role-playing episode, the assassination of a king is fictional, whereas hiding in a forest actually happened; (c) the awareness that a unicorn is a fictional animal, whereas a horse is a real one; (d) that beliefs about or attitudes toward real-world phenomena can be altered by exposure to fiction even when the fictional status is apparent—such as the belief that mental illness is contagious (as read in a novel) or the attitude that use of physical violence does not lead to serious injury (as seen in a cartoon).

### Separating memory of fact and fiction

According to our definition above, we see creating, using, recognizing, and remembering fictional information as distinct from factual information as a fundamental human cognitive capacity that spans several domains and situations. Fictionality has an evolutionary and ecological basis in the world-wide prevalence of children’s pretense (Gosso, 2010; Lancy, 2002), oral storytelling, and creative arts, such as literature and cinema. Children begin to pretend-play around the age of 12 months, and the pretense becomes more complex around the age of 3 to 5 years (Ma & Lillard, 2013). The ability to pretend-play may even predate humans (Ma & Lillard, 2017). Consequently, being able to distinguish between fact and fiction, and memories thereof, becomes critical for adaptive behavior. The reason is that memory of fact and fiction have different consequences for cognition and behavior. Remembered fact, in contrast to fiction, may adequately update subsequent attitudes, beliefs, and knowledge of the world and may be used for planning, decision-making, and goal-directed behavior. It stands to reason that actions are generally more adaptive when they are based on fact rather than fiction because they are more compatible with the real world. Notwithstanding, fictional contexts allow exploration of real ideas and social issues in a safe way. Just as previous research has suggested that the function of episodic memory is to allow simulations that can guide future behavior (Atance & O’Neill, 2001; Schacter et al., 2012; Szpunar, 2010), fiction has been argued to allow cognitive exploration (Dunk & Mar, 2022; Nissel & Woolley, 2022; Sugiyama, 2022) and to be particularly well suited to developing a social understanding of other people (Hutto, 2008; Mar & Oatley, 2008; Oatley, 1999, 2016).

Generally, people can separate fiction from fact in memory, but there are cases when the distinction is not upheld, and they mistake fiction for fact. A famous example is Orson Welles’s radio broadcast of *War of the Worlds* in 1938, which made many people believe that Martians were invading Earth (Bartholomew, 1998). Another example is a study by Fazio et al. (2013) in which people knew they were reading a fictional story but nonetheless later came to believe that “the Atlantic is the largest ocean.” Fiction can be persuasive and a potent source of misinformation: Studies have shown that beliefs and knowledge about the world can be influenced by fiction (Butler et al., 2012; Green & Brock, 2000; Rapp et al., 2014; Wheeler et al., 1999). However, generally, people behave and remember in accordance with the fictionality of the information as it was originally presented. Indeed, the regular confusion of fact and fiction would hinder understanding of the world and acting in it, as in pathological cases such as schizophrenia and delusional disorder (American Psychiatric Association, 2013). Consequently, a theory of the cognitive processing of fictional information needs to account for the distinction between fact and fiction that, on the one hand, is usually upheld, but on the other, is fallible and may result in people being misinformed by fiction.

Separating fact and fiction during on-line comprehension differs from separating memories of fact and fiction in several ways. One difference is that during on-line comprehension, the situational context and external cues, such as physical, social, and genre contexts, may guide the separation of fact and fiction. For example, opening a book labeled “Fairy Tales” will lead one to consider the contents as fictional, or the physical context of a theater will lead one to consider what happens on stage as fictional. By contrast, when remembering a piece of information, the physical or other contexts are usually not present. Another difference is that during on-line comprehension some pieces of information may be explicitly stated to be fictional. By contrast, when remembering this piece of information, the connected statement may not be available to help determine fictionality. Thus, determining the fictionality of information during remembering becomes a matter of memory processes to a larger extent than during on-line comprehension. The success or failure of such memory processes in determining the fictionality of information would be dependent on the memory’s associations and characteristics.

Comparative studies of memories of fact and fiction have indicated mostly similarities but also possible differences between the two. Memories of fact and fiction have been found to be fairly similar concerning phenomenological qualities. In one study, Hartung et al. (2017) studied reading of similar stories labeled as either fact or fiction and found no effect of fiction in memory on visual perspective or mental imagery. In a similar study design, Gander and Gander (2022) found no differences between memory of fact and fiction concerning clarity, visual detail, or first- and third-person perspectives (although location of third-person perspectives differed slightly). Further, Yang et al. (2022) compared a wide range of memory characteristics when people recalled autobiographical and fictional events. The results revealed a similar pattern for both conditions, although people rated fictional events slightly lower. In contrast, studies of other aspects of fiction processing have suggested differences between fact and fiction. Abraham et al. (2008) measured neural activation when participants processed sentences involving either real or fictional persons. For real persons, the neural activation seemed to indicate an involvement of episodic memory, whereas for fictional persons, semantic memory was more involved. We note, however, that these results are unclear, because there is a potential confounding: The stimuli used were such that the real persons may lead participants to visualize a realistic face, whereas the fictional persons may lead participants to visualize a face more like a cartoon. In another study of neural activation, Altmann et al. (2014) studied short stories labeled as either fact or fiction. They concluded that viewing the events as fact suggested a mental simulation of actions and their outcomes, whereas viewing the events as fiction suggested a stronger focus on what might have happened and the motives behind characters’ actions. However, the latter two studies focused on on-line comprehension, so the implications for memory processes remain unclear.

Overall, memory of fact and fiction seem to be fairly similar, and there is little to suggest that differences in the characteristics of the memories themselves can inform a mechanism that can help people separate the two when remembering. Rather, results of earlier studies point to a need for additional mechanisms beyond the content of the memory itself when determining the fictional status of a memory while remembering.

### The relation of memory of fictional information to other phenomena

We will now contrast memory of fictional information to some related phenomena to illustrate its relevance as a distinct construct. The similarities and differences are summarized in Table 1 in terms of four properties: *temporality* (i.e., whether past or future events are involved), *modality* (i.e., whether actual, possible, or fictional events are involved), *memory type* (i.e., whether semantic or episodic memory, or both, are involved), and *agent* (i.e., whether the self or another agent, or both, are involved). Focusing on these properties allows us to conclude that memory of fictional information is unique in that it refers to past-oriented fictional events that can involve both the self and others, and it can engage both episodic memory and atemporal, semantic information (presented at the bottom of Table 1). While examining the relation of fictionality in memory to other related phenomena, we evaluate to what extent existing theories could be applied to fictionality in memory. In the following, we argue that previous approaches have fallen short for several reasons: (a) They do not address fictionality but some other phenomena or concept; (b) they do address fictionality, but not in relation to memory, and they give no details that can inform our approach; and (c) they do not distinguish between fact and fiction in memory, and when they do, they do not provide an adequate conceptualization of fictionality.

**Table 1.** Comparison of Memory of Fictional Information to Some Related Phenomena

Phenomenon	Reference(s)	Temporality	Modality	Memory type	Agent
Episodic remembering	Tulving (1972)	Past	Actual	Episodic	Self
(Memory of) false information	Ecker et al. (2011)	Past	Actual-false	Semantic and episodic	Others
(Memory of) deception	Vrij (2015)	Past	Actual-false	Episodic	Others
(Memory of) fake news	Pennycook & Rand (2021)	Past	Actual-false	Semantic and episodic	Others
Mythical explanations of reality	Eliade (1968)	—	Actual	Semantic	—
Idealized scientific explanations of reality	Cartwright (1983); Suárez (2009)	—	Actual/possible	Semantic	—
Reality monitoring of internal versus external events	Johnson & Raye (1981); Johnson et al. (1988)	Past	Actual^a^	Episodic	Self
False memories	Loftus & Pickrell (1995)	Past	Actual^b^	Episodic	Self
Episodic future thought	Atance & O’Neill (2001); Szpunar (2010)	Future	Possible	Episodic	Self
Episodic counterfactual thought	De Brigard & Parikh (2019)	Past	Possible	Episodic	Self
Semantic counterfactual thought	Roese & Epstude (2017)	—	Possible	Semantic	—
Reported memories	Larsen & Plunkett (1987)	Past	Actual	Episodic	Others
Borrowed autobiographical memories	Brown et al. (2015)	Past	Actual	Episodic	Self
Vicarious autobiographical memories	Pillemer et al. (2015)	Past	Actual	Episodic	Others
Nonbelieved memories	Mazzoni et al. (2010)	Past	Actual-false	Episodic	Self
Event memory	Rubin & Umanath (2015); Rubin (2022)	Past	Actual	Episodic	Self and others
Memory of fictional events	Marsh & Yang (2020); Yang et al. (2022)	Past	Fictional^c^	Episodic	Others
(Memory of) pretense	Leslie (1987); Nichols & Stich (2000)	Past	Fictional^c^	Semantic and episodic	Self
Memory of fictional information		Past	Fictional^c^	Semantic and episodic	Self and others

Note: *Temporal* refers to events in the past or the future; *modal* refers to events that are actual, possible, or fictional; *actual-false* refers to events that are actual, but false; *semantic/episodic* refers to semantic or episodic memory, or both; *agent* refers to relating to the self or others, or both.

a Distinguishing real, external events from imagined, internal ones. ^b^As believed by the person who remembers. ^c^Decoupled from and not about the real world (see definition presented earlier).

First, we note that examples of phenomena that are excluded by our definition of fictional information are (memory of) false information, lies, idealized scientific explanations of reality, and fake news. These phenomena are different because they are intended to be about the real world or are associated with a communicative intention to deceive.

The reality-monitoring framework (Johnson et al., 1988; Johnson & Raye, 1981) explains how people separate memories of internal, imagined events (such as imagining locking the front door) compared to external, real events (such as actually locking the front door). According to this framework, memories of external events have more perceptual, spatial, and emotional details, whereas internal events have more traces of cognitive operations associated with the formation of the memory, such as thoughts and reflections. The memory system, instead of using a tag or label, attempts to classify a memory as internal or external on the basis of the memory characteristics, such as degree of perceptual detail. In some cases, reality monitoring could explain the separation of fact and fiction—or instance, distinguishing between reading about a fictional event compared to experiencing a real event. However, this does not hold generally, and even in the given example, it is a matter of reading versus experiencing, not between fact versus fiction. Indeed, both fact and fiction can come from mediated sources, such as text or moving images, with given perceptual details. Further, pretend play or role-playing takes place as external events just as much as everyday life events, and would be associated with similar patterns of perceptual details as real events.

The source-monitoring framework (Johnson et al., 1993; Mitchell & Johnson, 2009) describes the processes related to keeping track of the origins of information in memory: it includes both unconscious automatic processes acting on source information in memory and conscious more-elaborate processes of reasoning. In early work on comprehension of discourse, compartmentalization of fictional information from real-world knowledge in memory was attributed to links to the story source (Gerrig & Prentice, 1991; Potts et al., 1989; Potts & Peterson, 1985). Later work on the influence of fiction on attitudes, beliefs, and real-world knowledge characterized the process of attributing the fictional status of information as a source-monitoring process (Appel & Richter, 2007; Butler et al., 2012; Fazio et al., 2013; Green & Brock, 2000; Marsh et al., 2003; Rapp et al., 2014). We note that in this line of research, a methodology is used in which the fictionality of information is intertwined with the source of the information. However, in reality, a single source can convey both fictional and factual information, so fictionality cannot be decided on the basis of source identification only. One example is an educational children’s book including fantasy elements, which may convey both factual and fictional information. Additionally, in many cases, people can tell whether information is factual or fictional—for example, that unicorns and mermaids are fictional entities—without having any connection with a specific source. Finally, mere identification of a source does not account for how the cognitive system handles fictional information differently from factual information, and the consequences this has for cognition. Consequently, the source-monitoring framework can partly account for some cases of the fact-fiction distinction in memory—namely, when a correct source identification yields the fictional status of the information, but the framework is not sufficient to account for the phenomenon in its entirety.

Thinking of fictional events shares some properties with thinking about the future and what might have happened in the past but did not. One similarity is that memory of fictional events, episodic future thinking (Atance & O’Neill, 2001; Szpunar, 2010), and episodic counterfactual thinking (De Brigard et al., 2020) all involve events that did not occur. The key differences, however, are that simulations of the future and the past focus on possible events centered on the person (De Brigard et al., 2020; Jeunehomme & D’Argembeau, 2021; Szpunar, 2010). Fictional information, on the other hand, is about events that are decoupled from reality rather than possible events, and they need not be, and usually are not, centered on the person remembering. So far, research has focused on memory performance and phenomenological qualities of memories of past and future simulations (De Brigard et al., 2020; Jeunehomme & D’Argembeau, 2021). But it is unclear which mechanisms allow people to differentiate between past, imagined future events, and imagined counterfactual simulations, although reality-monitoring processes would be able to account for some of the distinctions.

Memory of fictional events differs from other phenomena described in relation to autobiographical memory. Memories of events reported by others (Larsen & Plunkett, 1987) and autobiographical-like events “borrowed” from other people (Brown et al., 2015; Pillemer et al., 2015) are all believed to be factual rather than fictional. Further, nonbelieved memories refer to vivid autobiographical memories of events that one once believed to have occurred but that are no longer seen as real in light of contradicting evidence (Mazzoni et al., 2010). We argue that no longer believing that an event occurred does not necessarily make it a fictional event. Although non-believed memories and memories of fictional events similarly evoke the belief that they did not occur, the intention and attitude to the memory is different. Whereas a nonbelieved memory would be considered false, a memory of a fictional event is encoded with the assumption that real-world truth conditions are irrelevant. It is an open question whether these two cases share important cognitive processes, but as we see it, in the case of memory of a fictional event, there is an additional connection to the concept of fiction.

A few memory models have been proposed that specifically address memory of fiction. Rubin and Umanath’s (2015) dimensional model of event memory and Rubin’s (2022) generalized dimensional model (which accounts also for semantic and implicit memory) both address memories of fictional events. Rubin proposed a conceptualization of memory using continuous dimensions as alternatives to the classical semantic–episodic (Tulving, 1972) and explicit–implicit distinctions (Squire, 1987). In Rubin’s model, memory is characterized in a conceptual space with three dimensions: explicit–implicit, possibility–impossibility of mentally constructing a scene, and self-reference or the lack of it. The dimensional properties extend throughout the conceptual space and combine to create locations. Considering examples of two typical types of memory, semantic memory would occupy the explicit non-scene construction, non-self-reference location, whereas memory of autobiographical events would occupy the explicit scene-construction, self-reference location. Rubin places memories of fictional events in the explicit scene-construction, non-self-reference location in the model. However, we note that this characterization does not distinguish memories of fictional events from memories of real reported events or vicarious memories told by others. This means that memories of events from, for example, a Harry Potter novel would be of the same type as memories of events reported in a newscast. This is unsatisfactory because we believe that the distinction between memory of fact and fiction has important consequences for cognition and behavior (see the section “Separating Memory of Fact and Fiction”). Because of these contrasting consequences, we see it as crucial that memory of fictional events is conceptually distinguished from memory of real events in a theory of human memory. Another approach by Marsh and Yang (2020) and Yang et al. (2022) uses a similar dimensional conceptualization with two dimensions, belief and agency, to account for memories of fictional events from movies, television, and novels. The theory aims to explain how memories of fiction, like autobiographical memory, can influence people’s view of themselves and their lives. However, this influence is considered only for explicit memories rather than for how fiction may influence people’s attitudes and beliefs in an unconscious way. In this theory, memories of fictional events are classified as memories that involve an agent other than the self and that are believed to have not actually happened—so the theory takes the important step of conceptualizing fictionality in memory. Yet we argue that the dimension of belief cannot fully capture the notion of fictionality. For instance, a vivid memory of an event initially believed to have happened can in light of new evidence be distrusted (nonbelieved memories; Mazzoni et al., 2010), but this does not make it a memory of a fictional event. Another limitation is that according to Yang et al.’s (2022) conceptualization, nonbelieved memories reported by others are classified as memories of fiction because they are believed not to have happened and because they do not involve the self. These types of memories may be rare, but we see no reason why they should be excluded conceptually. A further restriction is that memories of fiction can involve only others as agents. We argue that memories of pretend play or live role-playing activities could shift the agency from others to the self, even if the event is fictional. Finally, we make the general note that the conceptualizations of both Rubin (2022) and Marsh and Yang (2020) account only for memory of fictional events and lack an account for other types of fictional information, such as fictional objects, characters, or places and the ways these relate to other memory systems.

To summarize, earlier approaches do not adequately account for memory of fictional information. Many approaches concern related but different phenomena compared to memory of fiction concerning the time dimension, the modality of events involved, the involved agent, and the involved memory systems (summarized in Table 1). Further, the phenomenon of pretense falls clearly within our definition of fictional information (Nichols, 2006). However, earlier research on pretense has neither focused on memory nor exactly presented detailed mechanisms of how pretense and reality are kept separate (Leslie, 1987; Nichols & Stich, 2000), so the extent to which we can be informed by these approaches is limited. Accordingly, we conclude that the notion of fictionality cannot be accounted for by the reality-monitoring internal–external distinction, source monitoring, or belief whether an event occurred or not. Therefore, we claim that it is essential that models of memory specifically address the notion of fictionality (along the lines characterized by our definition) to account for memories of fictional information.

## A Framework for Memory of Fictional Information

The proposed framework has two main components. First, we show how our notion of fictionality can be integrated into a memory model. This step is mainly conceptual rather than process-oriented: We intend to clarify what researchers talk about when they talk about memory of fictional information. Second, we turn to the cognitive processes by describing a set of mechanisms that work to separate factual and fictional information in memory.

### Integrating fictionality in a memory model

We integrate the notion of fictionality into Rubin’s (2022) conceptual model of memory. At the same time, we acknowledge that fictionality may be incorporated into other memory models as well; Rubin’s model serves as one example. We see that it is particularly well suited for three reasons. First, it attempts to cover all types of human long-term memory and has overall good support from the memory-research literature. Stemming from the idea of covering event memory, it can account for memory of events not involving the self, which is something many memory models leave out (Rubin & Umanath, 2015). This becomes particularly relevant because much of fictional information comes from stories involving events that happened to others. Second, it is easily extendable with additional dimensions to account for fictionality. The properties of existing dimensions will also apply to any added dimensions, which provides a cohesive model. Finally, the idea to use continuous dimensions to account for basic memory properties is attractive. Fictionality of memory could then be seen as a matter of degree, reflecting a person’s graded belief (Dietrich, 2022).

Extending Rubin’s (2022) model with the fourth dimension of fictionality, we aim to account for the nature of memory of fictional information lacking in the earlier theories reviewed, which mainly applies to its decoupling from reality and one’s intention to engage in a creative act of imagination. The model can cover many types of memory of fictional information. It includes memory of fictional events but also fictional characters, places, and objects, which relate more to semantic memory than event memory. The added dimension of fictionality enables allocating the types of fictional information distinct from their factual counterparts within the conceptual model. Additionally, the model allows for both explicit and implicit memory of fiction.

**Fig. 1.**
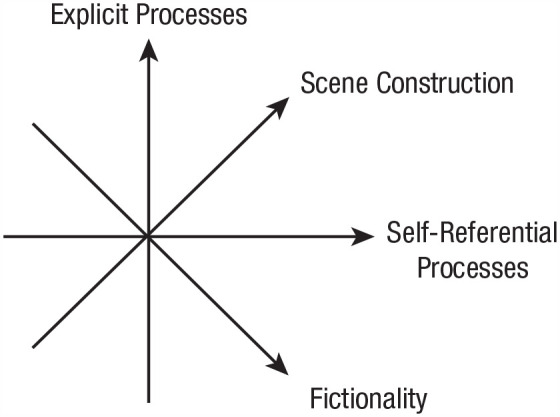
Dimensions of the memory model.

**Fig. 2.**
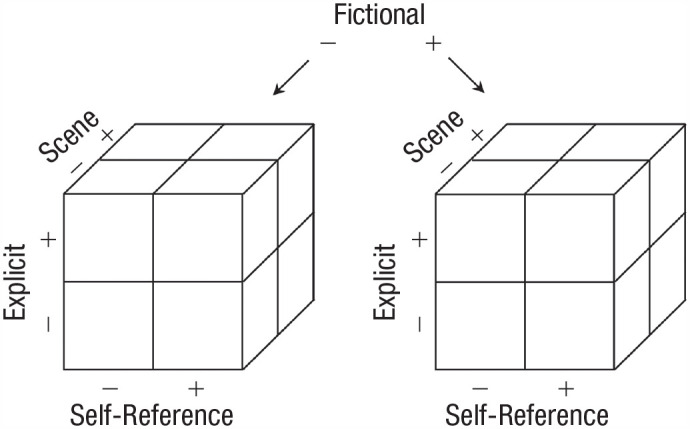
The dimensional space of the memory model divided into categories. The left cube corresponds to Rubin’s (2022) three-dimensional model; the fourth dimension of fictionality is depicted by an additional cube for memory of fiction.

The dimensions of the proposed model can be seen in Figure 1. Rubin’s original three dimensions are explicit processes, scene-constructing processes, and self-reference processes. The fourth added dimension of fictionality should be taken as orthogonal to the other three dimensions shown in Figure 1. In line with Rubin (2022), we assume for the moment that the continuous dimension of fictionality can be dichotomized into the two categories of fact and fiction. Adding this fourth dimension extends Rubin’s eight produced categories into 16 categories. The additional eight fictional categories mirror the original ones for fact (Fig. 2). We note that the continuous fictionality dimension could map onto the extent to which the rememberer believes the memory to be factual or fictional (Woolley, 1997). However, for the sake of clear communication, we use the dichotomized version of the model in the remainder of this article.

In the following, we will discuss the types of memory our model predicts, illustrated by the right cube in Figure 2. Table 2 presents these locations along with the types of memory that occupy them.^2^

**Table 2.** Types of Memory in the Locations of the Dimensional Combinations

Explicit	Scene+	Self+	Fact	Episodic and autobiographical memory
			Fiction	**Memories of events from role-playing, pretense, etc.**
		Self–	Fact	Vicarious and reported memories
			Fiction	**Memories of events from novels, movies, etc.**
	Scene–	Self+	Fact	Autobiographical facts and concepts
			Fiction	**Memories of concepts from role-playing, pretense, etc.**
		Self–	Fact	Semantic memory
			Fiction	**Semantic memory of fictional characters, objects, etc.**
Implicit	Scene+	Self+	Fact	Finding the way without experiencing anything, déjà vu
			Fiction	**Finding the way in a fictional world, e.g., video game**
		Self–	Fact	Same as Implicit, Scene+, Self+, “difficult to separate”
			Fiction	**Knowing the layout of a fictional world, e.g., a novel**
	Scene–	Self+	Fact	Personality, habitual ways of behaving, phobias
			Fiction	**Attitudes and influence on behavior from fiction on the self**
		Self–	Fact	Traditional laboratory studies of word lists, etc.
			Fiction	**Attitudes and influence on behavior from fiction or background information needed in, e.g., a mystery novel**

Note: *Scene+* and *Scene–* denote constructing a scene or not. *Self+* and *Self–* denote self-reference or non-self-reference. Descriptions of types of memory in the “Fact” rows are taken from Rubin (2022); boldface type indicates our additions.

### Categories of memory of fictional information

#### Explicit scene non-self-reference memory

Remembering events from fictional situations—novels, television, movies—employs memory that is explicit, for which a scene can be constructed, which does not involve the self, and which is about fictional rather than factual events. The fictional status differentiates this memory from vicarious memories (Brown et al., 2015; Pillemer et al., 2015) as well as all kinds of reported memories (Larsen, 1988; Larsen & Plunkett, 1987), such as news events and events from documentaries. Some enacted events, such as memory of events from a video game, would also belong here if the memory has little reference to the self—for example, by not involving bodily control of a player character or an avatar.

#### Explicit scene self-reference memory

We argue that in memory of fictional situations that involve actions, such as pretend play or live role-playing, there is some degree of self-reference, in addition to this type of memory being explicit and allowing scene construction. In these situations, the self (both physical and psychological) is not distanced from the fictional events. For instance, the fictional elements may share physical substrate with reality, so that an object, such as a real stone, is also a stone in the fictional world. The real action of throwing the stone could also correspond to pretending to throw an imaginary stone. In other words, the nesting of memories of fictional events within memories of real events, such as remembering the car ride to the movie theater to watch a movie (Yang et al., 2022), is blurred. This can shorten the distance between the real self and the represented self in the fictional situation, thereby placing the self in the imagined scene. Thus, memories of a fictional event, such as the role-playing of a workplace conflict, may contain strong references to the self.

Another case in which memory of fiction can have self-reference, even without enactment, is when the content is experienced as closely related to the self. For instance, the reader of a novel may strongly identify with the characters and the events, drawing parallels to the reader’s own life. Remembering events from the novel may then invoke a strong sense of self-reference, even though the self is not present in the imagined scene.

#### Explicit non-scene self-reference memory

One type of memory would be remembering concepts or states of affairs from pretense without remembering a scene—for example, the memory of pretending to be Superman and knowing that green kryptonite makes you weak.

#### Explicit non-scene non-self-reference memory

Explicit memories in which a scene is not constructed and in which there is no self-reference would correspond to the traditional notion of semantic memory (Tulving, 1972). Adding the fictional dimension to these memories would account for memories of fictional characters and objects. Memory of fictional places could be located in different positions in the model depending on its type. To the extent that the memory does not involve constructing a scene, that is, being semantic-memory-like (such as knowing facts about the Hogwarts School of Witchcraft and Wizardry from Harry Potter), memory of fictional places would belong to this part of the model. If the memory of a fictional place involves constructing a scene, such as imagining moving between different parts of Hogwarts, it is closer to memories of fictional events.

#### Implicit scene self-reference and non-self-reference memory

Being able to navigate in a fictional world in a video game without being aware of the spatial layout could be considered a type of implicit scene self-reference fiction memory. Similarly, implicitly knowing the spatial layout of a fictional world, such as from a novel, can guide narrative comprehension without involving self-reference.

#### Implicit non-scene non-self-reference and self-reference memory

Attitudes and beliefs adopted unconsciously from fiction can be characterized as implicit non-scene-constructing fiction memories. To the extent that these memories affect personality and habitual ways of behaving, they would be self-referencing—for example, fear of going into dark places after having watched a horror movie. Lack of self-reference would result in general attitudes originating from implicit memories of fiction, such as the idea that physical violence produces little harm, as in cartoons. This would offer an alternative explanation of the influence of fiction on attitudes and knowledge, which has been considered mostly at an explicit level as source-monitoring failures (Butler et al., 2012; Gerrig & Prentice, 1991; Green & Brock, 2000; Marsh et al., 2003) and impact of processing fluency (Fazio et al., 2013; Rapp et al., 2014).

Another type of implicit non-scene non-self-reference fiction memory can be background information from a story used in comprehension; one example might be clues about the identity of a murderer in a mystery novel.

### Mapping example cases onto the conceptual model

We now explicate how our conceptual memory model can be applied to the four previously presented examples of memory of fictional information. Considering the first example, remembering events from a novel about Harry Potter would employ explicit, non-self-referential, scene-construction fictional memory, whereas remembering characters and objects would employ similar but non-scene-construction memory. There may also be influences of implicit, non-self-reference memory, relating to scenes when understanding the layout of the fictional world in the novel. Similar implicit memory, but without scenes, may be operating when comprehending and reasoning about the plot in the novel. For the second example, remembering an episode of assassinating a king from a fantasy live role-playing session would involve explicit scene-construction, self-reference fictional memory. Turning to the third example, the awareness that a unicorn is a fictional animal whereas a horse is a real one would use explicit, non-scene-construction, non-self-referential fictional memory. Finally, in the fourth example, beliefs or attitudes altered by exposure to fiction would involve implicit non-scene-construction, non-self-reference fictional memory.

### Mechanisms and their hypothesized characteristics

**Fig. 3.**
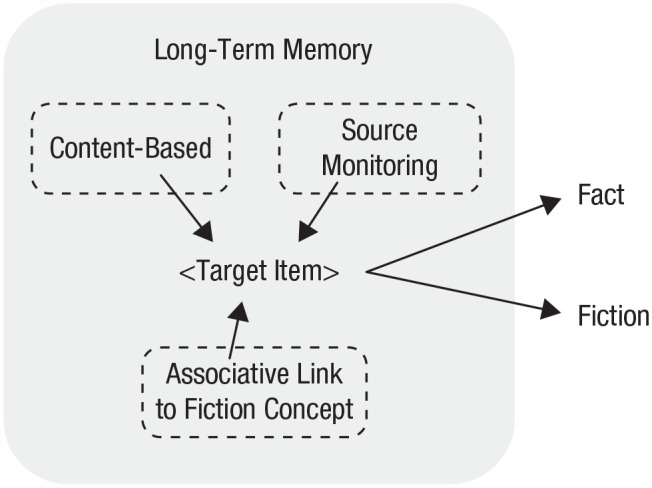
A target item retrieved from memory is classified as either factual or fictional using the proposed mechanisms.

Relying on previous research and the conceptual model outlined above, we describe three cognitive mechanisms underpinning the ability to separate fact and fiction in memory: content-based reasoning, source monitoring, and an associative link from the information to the concept of fiction (Fig. 3). Of these mechanisms, only source monitoring has been suggested in earlier research. In the following, we present the mechanisms and hypotheses concerning their key characteristics (Table 3). The hypothesized characteristics can be turned into predictions that can be tested in future research. Although these mechanisms are independent from one another, we acknowledge that more than one mechanism could operate simultaneously depending on the information available in the memory.

**Table 3.** Hypotheses of Mechanisms and Their Characteristics

Mechanism	Description	Characteristic					
		Processing level	Performance speed of separation	Cognitive resources taken mostly at . . .	Limited resources at encoding affects performance	Limited resources at retrieval affects performance	How does confusion of fact and fiction happen?
Content-based	The memory contents’ alignment with reality is used to evaluate the fictionality of the memory	Conscious	Slow	Retrieval	No	Yes	Content compatible with real world
Unconscious source monitoring	Automatic processes based on perceptual, contextual, and emotional details of the memory lead to identification of the source, followed by evaluating the fictionality of the source	Unconscious	Fast	Retrieval	Yes	No	Source misattribution
Conscious source monitoring	Content or contextual information leads to identification of the source, followed by evaluating the fictionality of the source	Conscious	Slow	Retrieval	Yes	Yes	Source misattribution
Associative link	An associative link is established to the concept of fiction	Unconscious	Fast	Encoding	Yes	No	Associative link is weak or missing

#### Content-based mechanism

The content of a memory could be a clue to whether it is factual or fictional. If the content of the retrieved memory is fantastical, impossible, or otherwise incompatible with reality, reasoning processes can conclude that this memory is not factual.

The content-based mechanism to separate factual and fiction information involves slow and conscious processing, which takes place entirely at the retrieval stage. The reason why the mechanism works only at retrieval is that the content of a memory is examined without any other information concerning its fictionality. In relation to the conceptual-memory model, the mechanism would use information stored in explicit non-scene non-self-referential fictional and factual memory. Limited cognitive resources at retrieval, such as when performing another concurrent task, could lower performance and increase errors in determining the fictional status. Limited cognitive resources at encoding would not affect the mechanism because it works on already encoded memories. Confusion between fact and fiction could happen with fictional content that is compatible with the real world: The mechanism would not enable separating fact and fiction in the case of realistic fictional content.

#### Unconscious and conscious source monitoring

The fictional status of the memory could be decided using source-monitoring processes in combination with a belief that the identified source is fictional (handled with an associative link; see the last mechanism below). The first part of this mechanism functions as proposed by the source-monitoring framework (Johnson et al., 1993), in which unconscious automatic processes utilize source information in the memory trace, and conscious reasoning processes use context to determine the origin of information.

In unconscious source monitoring, all types of implicit memory may be involved. These processes would work mostly at retrieval when they would be relatively undisturbed by limited cognitive resources. Limited resources at encoding could lead to weak memory traces of source information, which could introduce source misattribution errors. Misattributing some information from a fictional source to a factual source would lead to mistaking fiction for fact.

On a conscious level, source monitoring works more slowly and is based on explicit memory. The result of the source monitoring may be that a fictional source is identified, such as a specific novel. It may also be that during the monitoring processes, supporting memories are identified, such as the memory of the situation of encoding. For example, one may think back to sitting in a given room or armchair when reading that novel and in this way deduce that some piece of information is fictional (cf. nesting memories of fiction within personal memories; Yang et al., 2022). Another way source monitoring may enable identifying the fictional status is through a fictional context. For example, knowing that Hogwarts appears in the fictional story of Harry Potter would lead to a conclusion that it is a fictional establishment. Conscious source-monitoring processes work mainly at retrieval, and the performance of separating fact and fiction would be heavily reduced by limited cognitive resources at retrieval. A low level of encoded contextual information may also reduce performance.

Conscious source monitoring would fail to provide an accurate result of the fictional status of a memory if the source of the memory could not be identified. This could arise from weak or nonexistent source information. It could also happen after encountering information many times, as source information could be abstracted away, for instance, when something in episodic memory becomes semantic memory. Another case in which the mechanism would not be effective is when a source contains both factual and fictional information, so that it is not sufficient to correctly identify the source in order to determine the fictional status.

There is a difference between source-monitoring processes and content-based reasoning when determining whether a memory is of factual or fictional origin. Source-monitoring processes can lead to identifying the origin of the information, for example, a Harry Potter novel. This, in combination with an associative link from the Harry Potter novel and the concept of fiction, lets a person decide that the information is fictional. In contrast, using content-based reasoning, the fictional status is evaluated on the basis of the compatibility with the real world without identifying the source—for example, that flying brooms contradict reality and therefore must be fictional. Content-based reasoning would occur more when source information has not been encoded or when it has been reduced by abstraction as a result of multiple exposures. For example, people know that an event involving dragons is not real, but source information may be absent.

#### Associative link

People may still correctly determine the fictionality of information when neither the content nor the source of a memory provide reliable clues. How can this be explained? For example, adults know that Santa Claus is a fictional character. This is not because somebody with Santa Claus’s appearance would be impossible, and it is not because people remember the source from which they learned about Santa Claus. The knowledge that Santa Claus is fictional, we propose, is implemented as an associate link between the memory of Santa Claus and the concept of fiction. Being able to link Santa Claus to the fiction concept lets people adopt a view of Santa Claus as fictional—that is, that Santa Claus is decoupled from reality, that there is no point in searching for him in the real world, and that he is not subject to the same consequences as real people. Another example is that people know that a unicorn is a fictional animal. There is nothing physically impossible about a unicorn, and a specific source may not be available. The linking of the concept of a unicorn to the concept of fiction is what stops people from trying to book a unicorn safari or asking about the relation of unicorns to other animals in the phylogenetic tree of life. It would also prepare people to expect deviations from the real world when comprehending situations involving unicorns.

We conceive of such an associative link in a memory architecture, such as the network model of semantic memory originating from Quillian (Collins & Loftus, 1975; Collins & Quillian, 1969), but it may be implemented in other memory models as well. The associative link would be established unconsciously during encoding on the basis of a person’s belief that a certain piece of information is fictional. The content of the memory may be of any kind: explicit scene-constructing as memory of fictional events, or non-scene-constructing as in memory of fictional characters, places, and objects, but the link itself is handled implicitly. In this way, parts of an external source or situation may be encoded as fictional, whereas other parts are encoded as factual. This approach thus resolves the problem of classifying a source or situation as entirely factual or fictional when it actually has a mix of factual and fictional information.

Limited cognitive resources at encoding may lead to that the link is not established or is only weakly established. We hold that, by default, information is assumed to be factual unless indicated to be fictional (cf. Gilbert et al., 1993). Confusion between fact and fiction may then happen if the link is weak or missing, resulting in that fiction being taken as fact. This offers an alternative explanation of misinformation from fiction, which has previously been interpreted as source misattribution (Butler et al., 2012; Gerrig & Prentice, 1991; Green & Brock, 2000; Marsh et al., 2003; Potts et al., 1989; Potts & Peterson, 1985). Limited cognitive resources at retrieval would not influence the process as much because the process of association is assumed to demand few cognitive resources. We speculate that this mechanism—the ability to form such a link—would develop relatively early in childhood, because it relies only moderately on knowledge about the world and conscious reasoning. However, which information is linked to the fiction concept would vary greatly with age, because that relies considerably on knowledge of the world and what is real.

## Conclusion

Fictional information is ubiquitous in people’s lives through novels, movies, video games, pretense, role-playing, everyday conversations, and more. Memories of fictional information can affect behaviors, beliefs, and attitudes but need to be generally separated from factual information in order for people to successfully carry out planning, decision-making, and goal-directed behavior in a factual world. Therefore, as we see it, any general theory of human memory needs to take fictional information into account. In this article, we have argued that earlier approaches provide limited accounts in that they do not apply to memory of fictional information, and when they do, their analysis of fictionality is not adequate. To fill this gap, we have provided a theoretical framework of what memory of fictional information is and how fact and fiction are distinguished in memory.

First, we highlighted fictionality in memory as a distinct construct and a significant phenomenon to study. Second, we demonstrated how the notion of fictionality can be incorporated into a memory model by extending Rubin’s (2022) conceptual model of memory. As a result, our model can answer the question of what kind of memory the memory of fictional information is and show its relation to other kinds of memory. Explicit memories of fictional events, characters, places, and objects, as well as implicit memories of fiction, connect to different locations in the model. Dimensional combinations of our model propose new, unstudied areas of memory of fictional information for future investigation. Third, we have suggested a set of mechanisms that enable the separation of fact and fiction in memory. These mechanisms go beyond monitoring the source of information, which previous research suggested as the main process behind the representation of fictional information. Each mechanism explains how fact and fiction are separated in memory and under what circumstances fiction could be mistaken for fact, thereby offering an account of misinformation from fiction.

Our descriptions of these mechanisms provide hypotheses about their functioning in terms of consciousness, cognitive resources, and how confusion between fact and fiction happens. Future studies could test predictions from these descriptions. For example, a study could test the role of source information in memory of fictional information. The framework posits that source information in memory is not necessary in order to remember the fictional status of some piece of information. Participants could learn new factual and fictional information from several sources and then be tested on how well they remember the source and fictional status of these pieces of information. The framework makes several predictions: Participants’ performance on these two measures would be unrelated, and source information would not play a role to compartmentalize the fictional information. Instead, this is handled by a direct associative link according to the framework. Other studies could test predictions of the mechanisms concerning speed and demands for cognitive resources at encoding versus retrieval for confusion of fact and fiction in memory. For instance, increasing cognitive load by a concurrent task at retrieval would have detrimental effects for the slow content-based mechanism but would not influence the faster associative-link mechanism.

In addition to providing a conceptual and cognitive-processing road map for research on memory of fictional information, the framework has the potential to inform future research by reflecting on new questions related to the phenomenon of memory of fictional information. Therefore, we conclude by providing a nonexhaustive set of open questions raised by our work (Table 4).

**Table 4.** A Nonexhaustive List of Open Questions for Future Research

Category	Question
Model- conceptual	How could the dimensional continuous (i.e., noncategorical) values of fictionality be interpreted? Do they mirror the uncertainty of a person as to whether information is factual or fictional?
	What is the effect of single vs. multiple exposures for memory of fictional events, given that many fictional works allow reexperiencing whereas autobiographical events do not (see Yang et al., 2022)?
	Does the conceptualization of the self need to be more complex to account for memory of fiction, considering that in some fictional cases such as role-playing, there are at least two different selves (the physical self and the represented self) in the enacted fictional world?
	What is the conceptual relationship between memory of fictional and virtual experiences (e.g., using virtual or augmented reality technology)?
	Is it meaningful to consider how irregularly shaped spatial regions in the model map to types of memory?
Neurological	What key brain regions are involved in the mechanisms that separate fact and fiction in memory?
	What injuries or disorders can disrupt the capacity for separating fact and fiction in memory? What does this tell us about the neurological bases?
Emotions	Do emotions play a role (facilitating or hindering) in cognitive processing of fiction? Is it easier to remember the fictional status of highly emotional information?
Evolution	What evolutionary benefits does the capacity for fictionality have?
	How did the capacity for fictionality evolve?
	Do other animals also have this capacity?
	Is language a prerequisite for fictionality?
Development	When and how does the capacity for fictionality develop ontogenetically?
	Are there ontogenetic links between the capacity for fictionality and other capacities for engaging with nonfactual contents, such as theory of mind and counterfactual reasoning? If so, how are these capacities related?
Individual differences	Are there individual differences in the cognitive processing of fiction? Do differences depend on experience or more stable traits?
Culture	Are there cultural differences in the cognitive processing of fiction?
	Is there a relevant distinction between memory of reality, magic, religion, and fiction (see Boyer, 1997)?
Communication	What are the implications of cognitive processing of fiction for cases of misinformation and disinformation (e.g., fake news, propaganda)?

## Transparency

*Action Editor:* Mirta Galesic

*Editor:* Interim Editorial Panel

## Notes

1. It should be noted, though, that imagination (e.g., Liao & Gendler, 2020) is not unique to fiction and can involve factual information as well (Matravers, 2014), as in problem-solving or planning for the future (Atance & O’Neill, 2001).

2. Note that the conceptual model is not a taxonomy. Rather, the motivation to use a dimensional model is its advantages over a taxonomy; it is continuous, and properties combine across dimensions. What is presented in Table 2 is just one of several possible ways to structure the dimensional combinations.
